# Typology of Traumatic Events and Physical Function

**DOI:** 10.1093/geroni/igab046.3309

**Published:** 2021-12-17

**Authors:** Gabriella Dong, Mengting Li

**Affiliations:** Rutgers, The State University of New Jersey, New Brunswick, New Jersey, United States

## Abstract

Individuals experience various traumatic events over the life course, but little is known about the patterns of lifetime exposure to traumatic events. This study aims to identify traumatic event typology and examine its relationship with physical function. Data were from the 2017-2019 PINE study (N= 3,125). Traumatic events were evaluated by earthquake, typhoon, tornado, residential fire, physical assault, robbery, sexual assault, divorce, bereavement, cancer, homeless, imprisonment, and falsely accused. Physical function was measured by activities of daily living (ADL), with lower scores indicating better physical function. Analysis was conducted using latent class analysis and the four-class model fits the data best. We identified four typologies: limited trauma, severe trauma, natural disaster, and mild-to-moderate trauma. The “limited trauma” (33.8%) has the lowest exposure to all traumatic events except typhoon and homeless. In contrast, an equivalent “severe trauma” (33.3%) has the highest exposure to all traumatic events except natural disasters. A small “natural disaster” (4.8%) has the highest exposure to natural disaster and moderate exposure to other traumatic events. The “mild-to-moderate trauma” (28.2%) has mild-to-moderate trauma exposures. The mild-to-moderate trauma group (M=0.38, SD=2.12) has better physical function than limited trauma (M=0.69, SD=3.08), severe trauma (M=0.61, SD=2.81), and natural disaster (M=0.71, SD=3.22) groups. After controlling confounding variables, the mild-to-moderate trauma group has lower risks of ADL impairment than the limited trauma group (OR=0.66, 95%CI=0.47-0.93). The findings suggest mild-to-moderate exposure to traumatic events might benefit older adults’ health, while limited trauma might not be able to develop resilience and severe trauma overwhelms coping strategies.

